# Current Insights on Neurodegeneration by the Italian Proteomics Community

**DOI:** 10.3390/biomedicines10092297

**Published:** 2022-09-15

**Authors:** Tiziana Alberio, Martina Brughera, Marta Lualdi

**Affiliations:** Department of Science and High Technology and Center of Neuroscience, University of Insubria, I-21052 Busto Arsizio, VA, Italy

**Keywords:** neurodegeneration, proteomics, Italy

## Abstract

The growing number of patients affected by neurodegenerative disorders represents a huge problem for healthcare systems, human society, and economics. In this context, omics strategies are crucial for the identification of molecular factors involved in disease pathobiology, and for the discovery of biomarkers that allow early diagnosis, patients’ stratification, and treatment response prediction. The integration of different omics data is a required step towards the goal of personalized medicine. The Italian proteomics community is actively developing and applying proteomics approaches to the study of neurodegenerative disorders; moreover, it is leading the mitochondria-focused initiative of the Human Proteome Project, which is particularly important given the central role of mitochondrial impairment in neurodegeneration. Here, we describe how Italian research groups in proteomics have contributed to the knowledge of many neurodegenerative diseases, through the elucidation of the pathobiology of these disorders, and through the discovery of disease biomarkers. In particular, we focus on the central role of post-translational modifications analysis, the implementation of network-based approaches in functional proteomics, the integration of different omics in a systems biology view, and the development of novel platforms for biomarker discovery for the high-throughput quantification of thousands of proteins at a time.

## 1. Introduction

Neurodegenerative disorders (NDs) are complex diseases affecting a large part of the elderly population worldwide. As the average human lifespan is continuously increasing, the number of patients is expected to dramatically grow in the coming decades. Thus, studying the mechanisms involved in the pathobiology of NDs, for the development of novel disease-modifying therapies, represents an urgent unmet need, especially in terms of the impact on human society and economics.

The majority of NDs involve both genetic and environmental factors [1]. Familial forms—characterized by a known causative genetic alteration with classical mendelian inheritance—usually represent a small percentage (5–15%) of the total number of cases, while sporadic forms represent the majority of them. The latter often display high inter-individual variability, in terms of clinical manifestations, severity, and response to therapeutic treatments.

In this context, omics strategies are of pivotal importance for the identification of novel molecular factors involved in disease pathobiology, and for the discovery of disease biomarkers for early diagnosis, patients’ stratification, prognosis, and prediction of response to treatments [2,3]. These objectives are difficult to achieve, especially in the context of neurodegeneration research, due to many overlapping factors contributing to more than one disease, or simply related to ageing. The integration of omics data (genomics, transcriptomics, proteomics, metabolomics, meta-omics) represents the most important step towards the goal of personalized medicine.

The Italian proteomics community has been actively involved in the study of neurodegenerative disorders, by developing and using several proteomics approaches, which have contributed to changing the treatment of many complex neurological disorders during the last decade. Moreover, Italy is leading the Human Proteome Project initiative, which is dedicated to studying mitochondria as part of both chromosome-centric (c-HPP) and Biology/Disease (B/D-HPP) programs [4]. This is particularly important in view of the central role of the mitochondrial impairment in the pathogenesis of several NDs.

In this review article, we describe how the Italian proteomics community has contributed to the knowledge of many neurodegenerative disorders, in terms of (i) the identification of molecular factors and pathways central to the pathobiology of the disease, and (ii) the discovery of disease biomarkers.

## 2. Proteomics to Study the Pathobiology of Neurodegenerative Diseases

**Figure 1:**
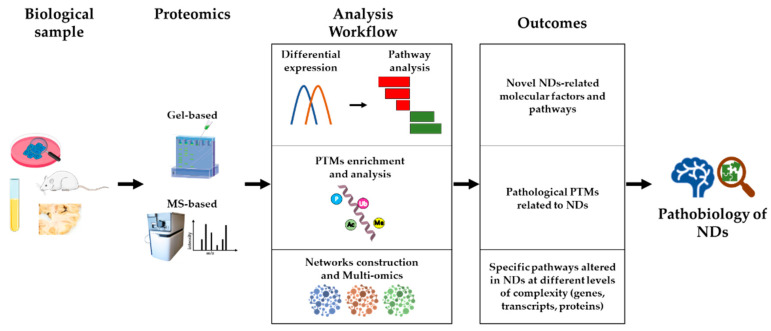
**Proteomics as a tool for studying NDs pathobiology.** Starting from several types of biological samples (cellular and animal models, biofluids, and patient-derived tissues), both gel-based and MS-based proteomics techniques can be used to investigate the pathobiology of NDs. Protein quantification, followed by differential expression analysis and pathway analysis, allows the identification of novel NDs-related molecular factors and pathways (see Section 2.1). Proteomics strategies have also been developed to enrich and analyze specific PTMs, leading to the identification of NDs-related protein modifications (see Section 2.2). Ultimately, the generation of networks of physically/functionally interacting molecules (proteins, transcripts, genes, metabolites) represents a tool for multi-level functional analysis, leading to the identification of specific pathways altered in NDs (see Section 2.3). PTMs: post-translational modifications.

Proteomics is playing a major role in research into the molecular mechanisms underlying the pathobiology of NDs, for several reasons. Firstly, proteomics strategies are untargeted, hypothesis-generating approaches, capable of unveiling novel factors and molecular pathways central to disease development and/or progression, which cannot be hypothesized *a priori*, based on previous knowledge. Secondly, proteomics allows not only high-sensitivity protein quantification and differential abundance analysis, but also the investigation of post-translational modifications (PTMs) that influence the activity and interactions of proteins, independently of their quantitative levels. Thirdly, starting from the output of a proteomics study (e.g., a list of differentially abundant proteins among experimental groups), a systems biology approach can highlight novel disease pathways and support targeted studies. Finally, integrating different omics data (e.g., transcriptomics, proteomics, and metabolomics), in the context of a multi-omics approach, could lead to a higher level of understanding of NDs biology, and be the most promising approach to achieve disease-modifying therapies for some NDs. An outline of this section is shown in Figure 1.

An emerging field, in which multi-omics approaches are becoming more and more important, is the study of the crosstalk between the central nervous system, the enteric nervous system, and the gastrointestinal tract—namely, the gut–brain axis. In this context, accumulating evidence suggests that microbiome dysbiosis (i.e., alterations in the optimal microbiome composition and activity) significantly contributes to the onset of several NDs. Indeed, metagenomics, metatranscriptomics, metaproteomics, and metabolomics have been extensively used to elucidate the modulatory effect of microbiota on the gut–brain axis, and its implications for neurodegeneration, using germ-free animal models, gut microbiota manipulation with antibiotics, and fecal microbial transplantation. These aspects are not debated here in more detail, having being recently reviewed elsewhere [5].

### 2.1. Proteomics as a Tool to Unveil Crucial NDs-Related Factors and Pathways

One crucial aspect in the study design of a proteomics experiment aimed at elucidating novel disease-related molecular mechanisms is the choice of the biological sample to be collected and analyzed. In general terms, cellular models (cell lines to perform in vitro assays), mouse models (knock-out mice for specific disease-causing genes; drug-treated mice), primary cells from patients (skin fibroblasts), iPSCs-derived neuronal cells, and affected tissues (autoptic frozen brains) are the most-used specimens for the mechanistic investigation of NDs pathobiology. On the other hand, biological fluids from patients (cerebrospinal fluid, blood, plasma, saliva, urine) are most frequently used for biomarker discovery. In this context, the choice of the specimen should not be underestimated, as it should mirror the disease state, whilst being invasive to the least degree possible. Cerebrospinal fluid (CSF) is likely the most common biological sample used to perform proteomics investigations in NDs. Indeed, despite being quite invasive for the patients, it effectively represents the wellbeing of the CNS, since many proteins and peptides coming from the CNS are found in CSF [6,7,8,9].

During the last decade, the Italian proteomics community has contributed to the elucidation of the pathobiology of several NDs, especially multiple sclerosis (MS), Alzheimer’s disease (AD), and Parkinson’s disease (PD), taking advantage of different experimental models and methods. The most recent discoveries are detailed below, and summarized in Table 1.

#### 2.1.1. Multiple Sclerosis

MS is a chronic immune-mediated demyelinating disease of the CNS, characterized by demyelinated lesions both in the white matter and the gray matter. It is a complex disease, with many genes which increase disease susceptibility, in addition to several environmental factors, e.g., Epstein–Barr virus infection, childhood obesity, and smoking. It is described as a two-stage disease, with early inflammation responsible for relapsing–remitting disease, and delayed neurodegeneration responsible for non-relapsing progression [19].

In this context, CSF has been widely used to monitor the inflammatory state and lesions extension. These two events might be correlated, as was inferred by Magliozzi and co-workers [10], who exploited an MS-based proteomics approach (i.e., TRIDENT, analysis for protein denaturation by three different protocols), and advanced 3T magnetic resonance imaging (MRI) to compare CSFs between control subjects and MS patients, stratified according to low (MSlow) or high (MShigh) levels of cortical lesions. They found: increased expression-levels of proteins related to the complement/coagulation cascade, to iron metabolism, and to the innate immune response; higher levels of sCD163, free hemoglobin, haptoglobin, and fibrinogen in MShigh patients; and higher levels of sCD14 in MSlow patients. Taken together, these data correlated the intrathecal dysregulation of iron homeostasis, the coagulation pathway, and macrophage activity with cortical damage onset and severity, for the first time.

Manconi and colleagues [11]—who exploited saliva as a biological sample for their study—performed an MS-based top-down proteomics approach, to investigate qualitative and/or quantitative differences in the salivary proteome and peptidome of MS patients. The use of saliva, for monitoring disease activity and response to treatment in MS, has many advantages; indeed, besides being a non-invasive diagnostic tool, it is a valuable representation of systemic health, as it contains plasmatic proteins transported from blood via intra- and extra-cellular pathways. From the analysis, proteins related to inflammatory processes and immune response were found differentially expressed in MS patients, compared to controls. Among these, cystatins, a superfamily of proteins involved in the downregulation of cysteine proteinases, were highly represented, suggesting the impairment of protein turnover and other antioxidant mechanisms.

#### 2.1.2. Alzheimer’s Disease

AD is the most common ND worldwide. It is described as a progressive form of dementia, whose main pathophysiological features are amyloid-β (Aβ) plaques and hyperphosphorylated tau protein accumulation, accompanied by elevated oxidative stress, reduced protein clearance, and a dysregulated immune response [20].

One recent discovery in AD pathobiology was the central role of astroglial alterations, not only in late gliosis and neuroinflammation, but also in the early stages of AD. In this context, Rocchio and co-workers [12] recently established a new model of immortalized astroglial cell lines from the hippocampus of a widely used AD mouse model (3xTg-AD) and wild-type (WT) control mice. At first, they used shotgun proteomics to confirm the astroglial phenotype and markers (glutamine synthetase, aldehyde dehydrogenase 1 family member L1, and aquaporin-4) and the maintenance of a proteomic profile compatible with that of primary astrocytes. Then, they applied a differential shotgun proteomics approach to unveil the differences between AD and WT immortalized astrocytes, highlighting calcium signaling, RNA binding, and ribosomal dynamics as novel pathways associated with astrocytes alterations in AD. Using the same cellular model, Dematteis and co-workers [13] demonstrated that bioenergetics was impaired in immortalized hippocampal astrocytes from 3xTg-AD mice, which displayed reduced glycolysis and oxygen consumption, and increased production of reactive oxygen species. The shotgun proteomics analysis of mitochondria–ER-enriched fractions showed no alterations in the levels of mitochondrial and OXPHOS proteins, while proteins related to ER functions and protein synthesis were deregulated. Still in the context of the role of astrocytes in AD pathogenesis, Tapella and co-workers [14] used a proteomics approach to demonstrate that, in mice with conditional deletion of calcineurin (downstream effector of intracellular calcium signals) in GFAP-expressing astrocytes, the hippocampus and cerebellum proteomes were deranged. The functional enrichment analysis revealed overrepresentation of annotations related to the myelin sheath, mitochondria, ribosomes, and the cytoskeleton.

Eventually, redox proteomics studies, performed by several Italian proteomics researchers, led to the identification of a ‘triangle of death’ in AD brains, which consists of the aberrant crosstalk among energy metabolism, mTOR signaling, and protein homeostasis [15].

#### 2.1.3. Parkinson’s Disease

PD is a movement disorder, and it is the second-most frequent ND worldwide, after AD. The hallmark motor symptoms are resting tremor, postural instability, bradykinesia, and rigidity, while non-motor symptoms include anosmia, cognitive dysfunction, sleep-awake dysregulation, dysautonomia, depression, and constipation. PD pathology is characterized by the specific loss of dopaminergic neurons in the substantia nigra pars compacta (SNpc), and the intraneuronal accumulation of protein aggregates, termed ‘Lewy bodies’. The etiology includes genetic susceptibility, aging, and environmental factors. Mitochondrial dysfunction, oxidative stress, dysproteostasis, and dopamine homeostasis alterations are well-recognized early pathogenetic mechanisms in PD, which trigger the detrimental events that lead to dopaminergic neurons death [21].

Neuroblastoma cell lines have been extensively used in PD research, as they represent an easy model to mimic early events in PD pathogenesis, especially mitochondrial dysfunction and altered dopamine homeostasis. These models have proven useful, not only for investigating PD pathobiology, but also for assessing the activity of candidate therapeutics [22]. Alberio and co-workers [16], using a model of altered dopamine homeostasis in neuroblastoma SH-SY5Y cells, demonstrated—by two orthogonal proteomics approaches (two-dimensional electrophoresis (2DE) and shotgun proteomics)—that the proteins affected by dopamine were related to the over-representation of gene ontologies associated with the generation of precursor metabolites and energy, the response to topologically incorrect proteins, and programmed cell death, thus linking altered dopamine homeostasis to mitochondrial damage. More recently—again using neuroblastoma cells as a model—Fazzari and co-workers [23] employed a proteomics approach, to demonstrate that the exogenous administration of ganglioside GM1 in Neuro2a cells increased the expression of proteins involved in mitochondrial bioenergetics. Moreover, the cells displayed an increased mitochondrial density and enhanced mitochondrial activity, together with reduced reactive oxygen species levels, thus suggesting the possible therapeutic potential of GM1 for the treatment of PD.

Another widely used cellular model, for the study of PD pathobiology, is primary skin fibroblasts derived from the skin biopsies of PD patients. These cells recapitulate, quite well, the events that occur in dopaminergic neurons, having the great advantage of easy handling in culture, and of minimal discomfort for the patient during sample collection [24]. Zilocchi and co-workers [17] recently investigated, by shotgun proteomics, total and mitochondrial proteome alterations in skin fibroblasts of *PARK2*-mutated patients, compared to controls. Both the network-based and the Gene Set Enrichment analyses of the differentially expressed proteins pointed out pathways in which Rab GTPases were involved. These proteins have recently emerged as central in the pathogenesis of both familial and sporadic forms of PD, thus highlighting vesicle trafficking alterations as a novel mechanism in PD pathobiology. Furthermore, Siciliano and colleagues [18] used a 2DE proteomics approach, to uncover alterations in primary fibroblasts from *PARK2*-mutated patients. As a result, they observed a reduced amount of N-terminal truncated proteolytic products of vimentin, a phosphoprotein belonging to cytoskeleton type III intermediate filaments, which has a major role in the maintenance of cell structure and integrity, cell adhesion, migration, and cell signaling. Collectively, these results highlighted the important role of impaired vesicle trafficking and cellular signaling in PD pathobiology.

### 2.2. Proteomics as a Tool for Investigating PTMs and Their Role in NDs Pathobiology

It is well-recognized that proteome complexity is much higher than that of the genome, since a single gene usually encodes much more than one functional protein. Even though the human genome contains about 21,000 protein-coding genes, the total number of proteins (i.e., proteoforms) in one cell is estimated to range between 250,000 and 1 million, depending on the cell type and the physiological state. In addition to this, proteins undergo dynamic synthesis and degradation, bind to the cell membranes, and interact with other proteins to form complexes [25]. The addition (and removal) of PTMs is crucial in generating diversity of proteoforms, sufficient to display different biochemical properties affecting protein structure and function, the ability to interact with other molecules, proneness to aggregate, and many other aspects of protein biology that can contribute to NDs pathobiology.

Recently, the role of PTMs in modulating protein function, and their centrality in pathogenetic mechanisms in neurodegeneration, have been extensively investigated by proteomics. Several methods, either gel-based or mass spectrometry-based, have been developed for the enrichment and quantification of specific PTMs in complex protein mixtures [26,27,28,29,30,31,32]. Some important examples of PTMs which play a role in NDs are: AMPylation—which was found to be involved in unfolded protein response and redox homeostasis [33]; tyrosine modifications (mainly oxidation)—which contribute to biological aging and age-related pathologies [34]; ubiquitination—the centrality of which is demonstrated by the fact that deubiquitinases are now recognized as potential therapeutic targets in neurodegeneration [35]; phosphorylation; methylation; nitrosylation; glycosylation; acylation; acetylation; succinylation; sulfhydration; and proteolysis, to name a few among more than 300 known possible protein modifications.

The Italian proteomics community has also contributed, in this field, to the understanding of the role of PTMs in NDs pathobiology, and to the development of methods for their efficient detection and quantification. The most recent discoveries are summarized in Table 2, and detailed below.

#### 2.2.1. Gel-Based Strategies to Investigate PTMs in NDs

Two-dimensional electrophoresis has been effectively applied to the study of PTMs, being a technique capable of discriminating between closely related proteoforms, which may differ by a single-residue modification. Indeed, proteoforms where modifications add or remove an electric charge will result in small changes in the net electric charge of the proteins, allowing them to be separated by 2DE. The analysis of PTMs usually combines 2DE protein separation with complementary biochemical assays that exploit specific enzymes or chemical reagents; alternatively, 2DE is combined with ex vivo experiments which are able to push the conditions that increase the PTM under study. Protein oxidative modifications, mainly, have been investigated using this approach, in AD and other NDs [43].

In this context, the investigation of multiple PTMs in AD has recently provided a more comprehensive view of all the dysregulated biological processes linked to the disease. The protein nitration (3-nitrotyrosine, 3-NT) profile was analyzed by Tramutola and co-workers [36] in blood CD3+ lymphocytes from AD patients. The rationale was that oxidative stress (both in the brain and at the periphery) influences the activation and differentiation of T-cells, the number of which was found to be decreased in the peripheral blood of AD patients. The authors performed immunoprecipitation of nitrated proteins, protein separation by 2DE, and LC–MS/MS identification of the differentially expressed nitrated proteins in AD compared with age-matched, non-demented subjects. Proteins involved in energy metabolism (PI3K and ATP synthase subunit alpha), cytoskeletal structure and intracellular signaling (ANXA2 and DRP2), protein folding and turnover (HSC71), and antioxidant response (CAT) were found to be more nitrated in AD. These altered pathways have been related to the reduced T-cell differentiation and altered immune response usually detected in AD development and progression.

Dysregulation in glucose metabolism has also been indicated as a hallmark of disease progression in AD and, interestingly, distinct research groups, examining different PTMs, have converged on the same results. Tramutola and colleagues [37] first investigated the link between AD and glucose metabolism dysregulation, as previous studies had already highlighted alterations of the hexosamine biosynthetic pathway (HBP) in AD patients. The end product of this pathway is UDP-N-acetylglucosamine (UDP-GlcNAc), the donor substrate for O-GlcNAcylation, a common PTM in neuronal cells, where the enzymes regulating O-GlcNAcylation are expressed up to 10 times more. The authors exploited a 2DE Blot proteomics approach to exploring alteration of the O-GlcNAcylation profile in 3×Tg-AD mice. As a result, the proteomics analysis highlighted many proteins with reduced O-GlcNAcylation levels, which belong to key pathways involved in AD, i.e., neuronal structure, protein degradation, and glucose metabolism. More specifically, the proteins affected were related to glucose metabolism (Gapdh, Eno1, and Mdh) and insulin signaling (IRS1 and Akt), less and more O-GlcNAcylated compared to WT mice, respectively.

The same authors also provided a more detailed view of the poly-ubiquitin profile in an AD brain [38]. This PTM is particularly important, both for signaling and for the maintenance of the proper protein homeostasis, by targeting proteins to degrade. One of the main pathophysiological features of AD is the accumulation of senile plaques composed by intracellular neurofibrillary tangles (NFTs), and extracellular plaques rich in amyloid-beta (Aβ). In this view, AD pathology is characterized by malfunction of the ubiquitin proteasome system (UPS), which is essential for removing oxidized, misfolded, polyubiquitinated proteins that can exacerbate the neurodegenerative process. Polyubiquitinated proteins were isolated from brain lysates by a high-binding affinity resin, then separated by 2DE, quantified, and identified by LC–MS/MS. Thirteen of the proteins displayed an increased poly-Ub profile in human AD brains, against controls. Among them, DRP2, HSP 90-β, and eIF2α showed an elevated poly-Ub on lysine 63 (K63), which is usually linked to protein degradation. These proteins are involved in axonal growth, protein quality control, and transcriptional control under stress conditions—processes that are already known to be impaired in AD. Notably, this was the first study to demonstrate alterations of the poly-Ub profile in human AD brains. The analysis of the same PTM on different disease-related targets could help to build a more robust and detailed network of altered connected proteins.

In the context of amyotrophic lateral sclerosis (ALS) pathobiology, Conti and co-workers [40] applied a differential proteomics approach to investigate modifications of the proteins of the skeletal muscle at disease onset. By differential shotgun proteomics, they found that the skeletal muscles of ALS patients showed increased levels of the myosin-binding protein H (MyBP-H), a feature that enabled the distinguishing of ALS patients from other motor neuropathies. MyBP-H interacts with Rho kinase 1 (ROCK1), whose pathway regulates the assembly of actin–myosin filaments, by phosphorylation of several targets, among which is the LIM kinase (LIMK). Given the increased expression of MyBP-H in ALS muscle, they investigated whether the downstream ROCK pathway was also affected: surprisingly, even though they detected an increased expression of ROCK in the muscle, the downstream signaling was impaired; indeed, treatment with lambda phosphatase revealed the accumulation of a non-phosphorylated LIMK1 in ALS patients. Moreover, the less acidic 2DE pattern of cofilin2 (a target of LIMK1) indirectly suggested that, downstream to LIMK1, cofilin2 was also less phosphorylated in ALS patients. In summary, the results of the untargeted differential proteomics analysis supported the execution of further hypothesis-driven experiments, which eventually demonstrated that, downstream to the overexpression of MyBP-H in ALS, there was a change in the pattern of PTMs (i.e., phosphorylation) of proteins involved in muscular actin–myosin regulation.

#### 2.2.2. MS-Based Strategies to Investigate PTMs in NDs

Mass spectrometry-based approaches are also widely used to investigate PTMs. The main advantage compared to 2DE is the higher sensitivity and wider dynamic range in protein detection and quantification. However, PTMs enrichment procedures are normally required for the application of MS-based approaches. To this end, the availability of specific antibodies for several PTMs allows for the efficient selection and enrichment, by immunoprecipitation, of modified proteins in complex protein mixtures. Alternatively, the biochemical properties of specific PTMs can be exploited, to perform a pre-fractionation of the protein sample (using either chromatographic or gel-based strategies), so as to obtain the enrichment of the modified proteins of interest.

PTMs analysis by MS was recently used to perform an in-depth investigation of the role of microglial cells in fostering neuroinflammation in the brain of AD patients, which is a central aspect in the pathobiology of the disease. In this context, Correani and co-workers [39] investigated the PARylated proteome of BV2 microglial cells treated with Aβ fragments. The rationale was that the CNS of AD patients presents an elicited inflammatory response and ROS production, which in turn provoke DNA damage that activates enzymes like poly(ADP-ribose)polymerase-1 (PARP-1). PARP-1 catalyzes the synthesis and the sequential attachment of multiple ADP-ribose moieties to a variety of target proteins; this PTM is named PARylation, and regulates downstream pathways such as transcription, chromatin remodeling, and DNA repair. After the treatment of BV2 cells with β-amyloid, and the enrichment of PARylated proteins by immunoprecipitation with the anti-PAR antibody, the authors identified the modified proteins via nano-LC–MS/MS: they were involved in mitotic control (SMC3, ANAPC4), ubiquitination (NEDD4 and USP10), and unfolded protein response/stress response (HSP70, DNAJC3, and TAO3 kinase). A mitochondrial enzyme—namely, the pyruvate dehydrogenase (PDH) subunit A—was found to be PARylated for the first time. Interestingly, PDH plays a key role in cellular metabolism, being the regulatory switch that couples glucose utilization to oxidative phosphorylation. In general, PARylation may represent one of the molecular switches responsible for the transition of microglial cells to an inflammatory phenotype, supporting the possible use of PARP inhibitors in the treatment of several NDs.

Protein sulfhydration also represents a crucial PTM in the context of several NDs. Greco and co-workers [41] have recently developed an analytical method for the accurate determination of hydrogen sulfide (H_2_S) in CSF. Depending on its concentration, H_2_S can act as either a neuromodulator or a neurotoxic factor, and plays a role in inflammatory and demyelinating disorders (acute disseminated encephalomyelitis, MS), in chronic NDs (AD, PD), and in motor neuron disease (ALS). The authors explored the low-molecular-weight proteome linked to sulfhydration by matrix-assisted laser desorption/ionization-time-of-flight mass spectrometry (MALDI-TOF MS). In particular, they obtained high-resolution protein profiling of transthyretin (TTR) isoforms related to supposed sulfhydration, as modifications of TTR in CSF are known to be related to CNS pathological conditions. Such modifications mainly occur in Cysteine residues, which are extremely sensitive to cellular redox balance, and represent the main targets of redox modifications. The authors’ results demonstrated that MS patients (being the patients who showed the highest levels of high-molecular-weight-bound sulfur species) showed a characteristic MALDI profile on TTR, and the levels of sulfhydrated TTR were significantly higher than those measured in other NDs. This was particularly important, because the modified TTR decreased its thyroxine-carrying activity, possibly contributing to the demyelination process in MS.

In the context of PD, Civiero and colleagues [42] discovered a new protein–protein interaction, of valuable interest for future PD-related therapeutic developments. PD pathology is often linked to mutations that enhance the activity of protein kinases: one example is the common G2019S mutation affecting LRRK2, whose overactivation causes decreased neurite length and complexity. Thus, protein phosphorylation is a PTM widely investigated in PD models. The authors analyzed the interactome of P21 (RAC1)-Activated Kinase 6 (PAK6), a previously identified binding partner of LRRK2, demonstrating a direct interaction with a subset of 14-3-3 proteins in a kinase-dependent manner. After phosphopeptides enrichment and LC–MS/MS analysis, they observed that PAK6 bound and phosphorylated the adaptor protein 14-3-3γ, causing the dissociation of the chaperone from LRRK2, and subsequent LRRK2 dephosphorylation. Interestingly, they demonstrated that a constitutively active form of PAK6 was capable of rescuing the G2019S LRRK2-associated neurite shortening through phosphorylation of 14-3-3γ. Collectively, these data support the possibility of targeting phosphorylation as a therapeutic strategy.

### 2.3. Network-Based Functional Proteomics and Integrated Omics to Investigate NDs Pathobiology

One shared feature of all omics strategies is that the final outcome is a huge matrix of complex data, which allows the researchers not only to identify a panel of ‘significant’ molecules (also by means of a feature-selection procedure), but also to investigate the functional relationships among them (e.g., the involvement in a specific pathway), thus generating interaction networks and new working hypotheses [44]. Indeed, due to their complexity, omics datasets can be effectively represented and analyzed in the form of networks, where nodes represent the observations (proteins, genes), and edges represent the associations among them.

**Figure 2:**
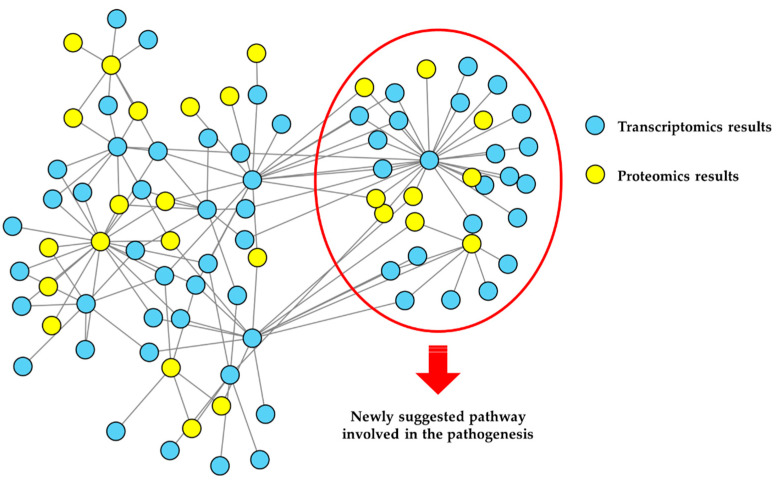
**An example of network-based functional proteomics and integrated omics, used to gain new insights into NDs pathobiology**. Results (e.g., gene products differentially expressed) coming from different omics strategies can be used to build a network model of the ND under investigation. Edges may represent, as in this case, protein-protein interactions (PPIs). Various analyses, such as over-representation analysis after network clustering, can provide a functional interpretation of the results. In this example, proteins differentially expressed (blue nodes) or differentially abundant (yellow nodes), as observed by transcriptomics and proteomics experiments, are first interactors and strictly interconnected, suggesting that they take part in the same biochemical pathways. Evidenced nodes are involved in the same process (over-represented), suggesting their involvement in the ND pathobiology.

The combination of quantitative omics strategies with network-based analysis is at the basis of the concept of network medicine [45]. A multifactorial approach, which is characteristic of network medicine, is required, to investigate the multiplicity of factors that can alter a complex system, and to identify relationships with the clinical phenotype [46]. This can be done at increasing levels of complexity by the integration of datasets obtained from different omics approaches—for example, proteomics and metabolomics data—through the use of specific bioinformatics workflows [47,48]. A graphical representation of the network-based functional analysis of omics data and their integration is shown in Figure 2.

#### 2.3.1. Network-Based Strategies to Identify Specific Altered Pathways in NDs

Italian proteomics researchers have recently contributed to the definition of novel disease mechanisms in neurodegeneration, through the use of network-based functional analysis.

Monti and co-workers [49] used a network-based approach to investigate unique and shared mechanisms in neurodegeneration, by comparing PD, AD, and ALS. The authors performed a meta-analysis of the literature, gathering all quantitative proteomics investigations, in different models of the three diseases. Then, matching proteomics and genetics data, they identified 25 proteins uniquely involved in PD, and validated increased levels of transaldolase in SNpc samples from five PD patients. From network-based functional analysis, the biological processes specifically affected in PD proved to be proteolysis, mitochondrial organization, and mitophagy, thus confirming mitochondrial dysfunction as a specific and central event in the pathogenesis of PD.

Recently, Cozzolino and co-workers [50] performed an immuno-affinity purification-mass spectrometry (IP-MS) study of endogenous ADAM10 in the brains of wild-type and Huntington’s disease (HD) mice. In physiological conditions, proteins implicated in synapse organization, synaptic plasticity, and vesicle trafficking interacted with ADAM10, suggesting that it may have acted as a hub protein at the excitatory synapse. The authors analyzed the interactome of ADAM10, which proved to be enriched in presynaptic proteins. Then, using a network-based functional enrichment analysis, they identified ADAM10 and its interactors as key players in the recycling and maintenance of an active pool of synaptic vesicles, an impaired process in HD pathogenesis.

Although the main application of network-based approaches is the identification of disease-specific protein networks, another complex aspect of NDs that can be investigated by omics approaches and network construction is neuroprotection. Indeed, the possibility to counteract neurodegeneration by the strengthening of endogenous protective mechanisms against cellular stress is very attractive for the development of new therapeutic strategies against the progression of many NDs. Very recently, thanks to the use of high-throughput proteomics techniques (redox proteomics, in particular), a network of genes encoding proteins (called ‘vitagenes’) involved in neuroprotection has been discovered [51]. Vitagenes are protective genes which control the activation of pro-survival pathways, anti-oxidant, and anti-apoptotic factors, in response to cellular stress. In this context, Dattilo and co-workers [52] unveiled heat shock proteins as prominent members of the vitagene network, and further highlighted the importance of redox proteomics as a tool to investigate the redox modulation of stress-responsive vitagenes, to favor neuroprotection.

#### 2.3.2. Integration of Different Omics Strategies

The Italian proteomics community has also contributed to the development of strategies aimed at integrating different omics data into the holistic realization of a ‘systems biology’ understanding of NDs. The so-called ‘integrated omics’ strategy—also referred to as ‘multi-omics’ or ‘pan-omics’—aims to integrate multi-dimensional omics data into a biologically meaningful context. To do so, individual omics datasets that are closer to genotype (genomics and transcriptomics), and those that are closer to phenotype (proteomics and metabolomics), are integrated, using statistical or advanced machine-learning approaches, which represent the most challenging step [53]. The results may be simple pathways or complex networks, and might eventually be exploited to predict health or disease states, provide insights for effective therapeutic interventions, and reveal spatiotemporal regulation of biological systems. Recently, Hampel and co-workers [54] described how omics sciences (genomics, epigenomics, transcriptomics, proteomics, metabolomics, and lipidomics), embedded in a systems biology framework, are capable of generating explainable readouts describing the entire biological continuum of an ND. More specifically, they highlighted how multi-omics data, integrated into systems biology network approaches, can detect upstream pathological alterations and downstream molecular effects occurring in pre-clinical stages. Some examples of such pathways are apoptosis, synaptic vesicle trafficking, neurite outgrowth, lipid homeostasis, oxidative stress, coagulation, energy metabolism, and mitochondrial dysfunction.

Considering the different levels of complexity of omics data, that of metabolomics is widely recognized to be the highest. Metabolomics—which is the actual omics approach to investigate phenotypic differences [55]—is the study of metabolites, defined as all bioactive small molecules (<2000 Da) in a biological sample; they are considered to be the result of multiple interactions between genes, transcripts, and proteins [56]. Amino acids, lipids, sugars, and steroids are some examples of metabolites. The aim of a metabolomics study is the comprehensive and simultaneous determination of the levels of virtually all metabolites in a sample.

This strategy was used by Stella and co-workers [57] during the evaluation of changes in both the cellular proteome and the secreted metabolome of primary spinal cord astrocytes derived from a widely used ALS mouse model, which overexpresses the human mutated (h)SOD1(G93A) protein, compared to (h)SOD1(WT) mice. The aim of the study was to discover the mechanisms that promote astrocytes failure and hyperactivation, contributing to neuro-inflammation and motor neurons death in ALS. Untargeted metabolomics analysis was performed, using a conditioned medium from cultured primary astrocytes, while a labeled shotgun proteomics approach (6-plex TMT) was used to quantitatively analyze the proteome of the astrocytes. Glutathione (GSH) metabolism proved to be significantly affected, as glutathione–cysteine ligase (the rate-limiting enzyme involved in GSH biosynthesis) and γ-glutamil-tyrosine (involved in GSH metabolism) were reduced in (h)SOD1(G93A), compared to WT. Moreover, alterations of lipid metabolism emerged, with some free fatty acids over-represented in hSOD1(G93A) astrocytes, and decreased abundance of phosphatidic acid. Eventually, the authors found alterations in the ubiquitin–proteasome and endosome–lysosome systems, with higher levels of the regulatory subunit 8 of the 26S proteasome (PSMD8) and lysosomal cysteine proteases (cathepsin B and cathepsin Z). This was explained by the observed increased activation of some transcription factors (i.e., Nf-kB, Ebf1, and Plag1), associated for the first time with ALS pathology.

As a general comment, it is worth noting that integrated omics represents progress towards gaining novel insights into the pathobiology of NDs. These achievements will suggest new targets and support the design of effective (and possibly personalized) therapeutic strategies.

## 3. Proteomics to Discover Disease Biomarkers for NDs

Proteomics has been extensively used for biomarker discovery in neurodegeneration, and there is plenty of scientific literature suggesting panels of proteins that should work as either diagnostic/prognostic indicators or as biomarkers for patients’ stratification and drug-response prediction. The use of unbiased omics strategies is needed, to analyze and quantify thousands of features and, eventually, to extract reliable disease-associated markers [47]. This requires the application of stringent feature-selection workflows, to highlight relevant features (e.g., proteins) and multivariate statistical analysis, to identify panels of markers that can discriminate among subject groups and/or predict specific outcomes.

**Figure 3:**
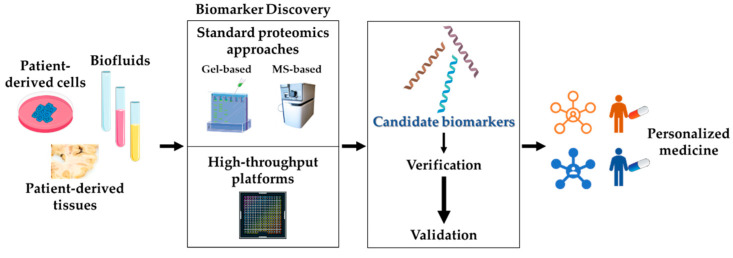
**Biomarker discovery by proteomics.** Proteomics represents the most promising approach to the identification of reliable disease biomarkers, both in the central nervous system and at the periphery. The discovery phase starts with the choice of the appropriate biological sample (patient-derived biofluids, cells, and tissues). The proteomics analysis is then performed, either by standard methods (see Section 3.1) or by newly developed, automated high-throughput platforms (see Section 3.2). Candidate biomarkers derived from the discovery phase are then subjected to verification and validation processes. Validated molecules will: (i) work as diagnostic/prognostic indicators; (ii) allow patients’ stratification; (iii) predict individual drug response; key steps towards personalized medicine.

In the context of NDs biomarker discovery, classical gel-based and MS-based approaches have been used in proteomics for the analysis of biofluids, peripheral tissues/cells, and autoptic brains. Currently, novel high-throughput platforms are being developed to speed up the process, to standardize procedures, and to increase the sensitivity (Figure 3).

### 3.1. Gel-Based and MS-Based Approaches to Biomarker Discovery in NDs

Italy-based proteomics studies have been contributing to knowledge in the field of NDs biomarkers for the past decade.

In the context of PD, Alberio and co-workers [58] analyzed the proteome of T-lymphocytes from PD patients, and matched the control subjects by 2DE. In terms of protein levels, they reported β-Fibrinogen levels to be lowered in PD patients, whereas some heavy isoforms of transaldolase were more abundant. The authors also built predictive models, verified by leave-one-out cross-validation, and proposed two functions capable of staging PD subjects. They identified a panel of seven proteins showing different levels in early-onset, with respect to late-onset PD patients. Then, the authors performed a verification step, in which the panel of proteins was quantitatively assessed by multiple reaction monitoring (MRM) in T-lymphocytes from PD patients and controls [59]. The discriminant function obtained from the 2DE analysis was used to classify the patients, while protein amounts were measured by MRM. A good discriminant function was obtained both by selecting a subset of peptides and by using all peptides in the selected panel of proteins. However, a large-scale clinical validation is needed, to corroborate the results and the methods.

Similarly, Baldacci and co-workers [60] recently highlighted the role of YKL-40, an indicator of microglial activation and neuroinflammation, as a candidate biomarker for AD. Indeed, YKL-40 concentration in CSF has been shown to predict the progression from prodromal mild cognitive impairment to AD dementia. In addition, a positive association between YKL-40 and total tau protein levels in CSF was reported during the asymptomatic pre-clinical stage of AD. Thus, YKL-40 represents a candidate biomarker, mirroring distinct molecular mechanisms in the brain that can be useful for patients’ stratification for the targeted anti-inflammatory therapies now emerging from precision medicine.

Despite many efforts, the success rate of most panels of biomarkers, in supporting clinical trials over the last ten years, has been extremely low [61,62]. The explanation resides in many possible pitfalls in biomarker research workflows, such as the choice of the specimen (e.g., brain tissue, plasma, serum, cerebrospinal fluid), the quality and number of the samples and their respective controls, the sensitivity of the quantitative proteomics approach employed, the robustness of the statistical analysis performed, and the choice of an effective validation. All possible pitfalls in biomarker discovery by proteomics have been recently summarized by Nakayasu and co-writers [63], who report that low numbers of samples and lack of proper validation are the major challenges. Moreover, successful biomarker studies always involve multidisciplinary teams of clinicians, analytical chemists, and statisticians, highlighting the importance of gathering together different skills to conceive an appropriate study design. Finally, technology improvements are needed, and will, for sure, have a major impact; in this context, multiplexing with isobaric tags, faster chromatography, and additional separation techniques (e.g., ion mobility spectrometry) have the potential to dramatically increase the speed and reduce the cost of analyses.

Related to the choice of the most appropriate specimen for performing biomarker discovery in the context of NDs, Pieragostino and co-workers [64] recently published a study in which the content of extracellular vesicles (EVs) from tears was analyzed in MS patients by shotgun proteomics. Tears represent an interesting matrix for biomarker research, because they are related to the CNS, and contain EVs derived from neuron cells. The authors sorted microglia-derived and neural-derived EVs from the tears of MS patients, and compared their protein content to that of EVs purified from CSF. They demonstrated that EVs from both CSF and tears conveyed the same protein groups involved in inflammation, angiogenesis, and immune response signaling. These results support the existence of a molecular crosstalk between CSF and tears, which opens up the possibility of exploring tears as a biological sample to be used for biomarker discovery in several NDs.

Of note, biomarker research in the field of NDs is also complicated by the fact that several biochemical pathways are altered the same way in different NDs, and this hampers the identification of single (or a panel of) biomarkers that are unique to a specific ND. Nevertheless, the use and integration of omics strategies and newly developed neuroimaging techniques [65] still represent the best choice for the discovery of reliable early biomarkers, especially those that are capable of identifying the so-called preclinical (or prodromic) stage of NDs [66,67,68]. Indeed, it has been clearly demonstrated that extensive pathological changes in the brain occur many years before disease diagnosis, which represents a temporal window for the effective application of both neuroprotective and disease-modifying therapies.

### 3.2. High-Throughput Platforms for Biomarker Discovery in NDs

Italy-based proteomics studies also contributed to biomarker research in NDs, through the use of recently developed platforms that allow for the identification and quantification of thousands of proteins in biological fluids. SomaScan^®^ technology [69] was used by Morani and co-workers [70] in the context of autosomal recessive spastic ataxia of Charlevoix–Saguenay (ARSACS). Although this rare disease is known to be caused by mutations in the sacsin gene, the mechanisms contributing to the disease have still to be clarified, and no therapeutic strategies are currently available, nor any early disease biomarkers. The authors were able to obtain an accurate proteomic profiling of skin fibroblasts from healthy controls and ARSACS patients, and of WT and sacsin knock-out SH-SY5Y cells. The major differentially expressed proteins (DEPs) found were related to neuroinflammation, synaptogenesis, and engulfment of cells, providing the basis for future studies.

Another interesting application of this quantitative assay was reported in the context of AD. Indeed, very recently, some Italian researchers contributed to an international study aimed at discovering and replicating plasma proteomic biomarkers related to AD [71]. The National Institute on Aging and Alzheimer’s Association (NIA-AA) proposed, in 2018, some guidelines (termed ‘ATN’) for classifying AD based on biomarkers of amyloid pathology (A), tau pathology (T), and neurodegeneration (N) [72]: ‘A’ is measured by cortical amyloid PET ligand binding or CSF Aβ-42; ’T’ is measured by CSF phosphorylated tau or cortical tau PET ligand binding; and ’N’ is determined by CSF total tau, 18F-fluorodeoxyglucose PET, or brain atrophy on magnetic resonance imaging. The main issue with these measures is that they are challenging, because of their invasiveness, high cost, and limited availability. Blood-based biomarkers, on the other hand, could represent a less invasive and cost-effective option for the detection, classification, and monitoring of AD. In this regard, the authors analyzed plasma proteins from 972 subjects (372 controls, 409 mild cognitive impairment [MCI], and 191 AD) using both the SomaScan platform (4001 proteins quantified) and targeted assays (ELISA and Luminex; 25 proteins assessed). Using hub proteins resulting from co-expression and differential expression analyses, age, and apolipoprotein Eε4 genotype, they were able to discriminate AD from controls with an area under the curve (AUC) of 0.81, and to discriminate MCI convertors from non-convertors with an AUC of 0.74. Collectively, the study clearly suggests that blood proteins can predict the presence of AD pathology. Of note is the application of both proteome-wide differential analysis and protein co-expression network analysis, suggesting new insights into changes in individual proteins and protein networks linked to AD.

Collectively, the growing development of high-throughput platforms for protein biomarker discovery represents the best chance of overcoming all the limitations and pitfalls mentioned above, possibly leading to the discovery of reliable biomarkers for NDs.

## 4. Mitochondrial Proteomics and Neurodegeneration

Neurons are terminally differentiated cells which fully rely on oxidative phosphorylation for their energy supply. Healthy mitochondria guarantee not only ATP production, but also the maintenance of a correct metabolic homeostasis, the regulation of Ca^2+^ fluxes and redox signaling, and the arbitration of synaptic activity and cell survival [73].

**Figure 4:**
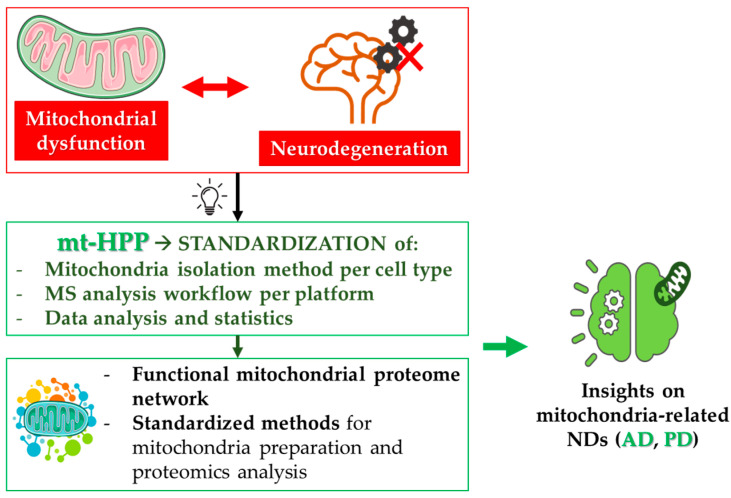
**Mitochondria and neurodegeneration.** Mitochondrial dysfunction represents one of the most frequent pathological alterations in NDs. Indeed, the failure of the tightly regulated mitochondrial quality control contributes to neuron death. The mitochondria-centered initiative of the Human Proteome Project (mtHPP), which aims at the in-depth investigation of the mitochondrial proteome in health and disease, has enabled the standardization of procedures related to mitochondria isolation and quantitative analysis of mitochondrial proteins (see Section 4.1). The fulfillment of the objectives of this project will achieve important advances in the understanding of several NDs, especially AD and PD (see Section 4.2 and Section 4.3, respectively).

Mitochondria are unique organelles endowed with their own genome, the mitochondrial DNA (mtDNA), encoding thirteen mitochondrial proteins of the electron transport chain (ETC) [74]; thus, mitochondrial activities are orchestrated by both nuclear and mitochondrial genes. Moreover, mitochondria are not standalone entities; rather, they are organized in complex, continuously remodeled networks, that allow them to exchange components and to dispose dysfunctional organelles. Network dynamics are mainly regulated by fusion and fission processes, which collectively represent the mitochondrial quality-control machinery [75,76,77]. When the mitochondrial quality control fails, and dysfunctional mitochondria are not properly eliminated, low ATP production, in combination with the generation of reactive oxygen species, leads to increased oxidative stress. This causes dramatic changes in cellular homeostasis, which can trigger cell death. In neurons, these mechanisms initiate and/or foster neurodegeneration. As demonstrated by a literature meta-analysis [49], the degeneration of synapses, led by mitochondrial impairment and metabolic failure, can be the starting event in these neurodegenerative processes. For this reason, mitochondrial dysfunction is well-recognized, and has been deeply investigated as an early event in the pathogenesis of several NDs, especially AD and PD [78,79]. In this frame, the efforts of the Italian mt-HPP initiative helped the identification of novel mechanisms in the crosstalk between mitochondrial dysfunction and neuronal cell death in NDs (Figure 4).

### 4.1. The Italian mt-HPP Initiative

As mentioned above, the mitochondrial Human Proteome Project (mt-HPP) is a Human Proteome Organization (HUPO) initiative led by the Italian Proteomics Association (ItPA) (https://hupo.org/mitochondria). The main goal of this effort is to deepen the integrative role of proteins acting at the mitochondrial level, considering both those coded by the mt-DNA and those coded by the nuclear genome. The specific aims of this initiative have been: (i) the definition of the mitochondrial proteome by the characterization of all mitochondrial or mitochondria-associated proteins and their interactions in health and disease [80]; (ii) the definition of a functional mitochondrial proteome network; (iii) the development of standardized methods for mitochondria preparation and proteomics analysis; (iv) the characterization of the mitochondrial proteome/interactome in both neurodegenerative and metabolic diseases; (v) the identification of three missing proteins in the mitochondrial proteome. The achievement of these aims will dramatically increase our knowledge of mitochondrial biology, in both physiological conditions and disease states.

In the context of the mt-HPP, the Italian proteomics community has contributed to investigating the role played by mitochondrial proteins in the initiation and progression of neurodegenerative processes: multicentered experiments have been performed, using different MS platforms and different cellular models, to establish standardized protocols for mitochondria proteome analysis [81].

Furthermore, novel strategies have been proposed and optimized for the characterization of the mitochondrial proteome. The most important example is redox proteomics, which is that branch of proteomics used to identify oxidized proteins, and to determine the extent and location of oxidative modifications in proteins of interest. The steep increase in the development of redox proteomics strategies, pioneered by Perluigi and co-workers, has allowed researchers to unveil neurodegeneration-related changes in protein expression linked to protein oxidation levels [82,83]. This kind of approach is especially important in the study of NDs, where protein oxidation is a frequently observed pathobiological event.

### 4.2. Mitochondrial Proteomics in Alzheimer’s Disease

The seminal work of Castegna and co-workers suggested, for the first time, a relationship between protein oxidation and protein abundance, thanks to redox proteomics studies in AD brains [84]. They demonstrated that the targets of protein oxidation tend to precipitate as insoluble matter, suggesting an inverse relationship between protein oxidation and the amount of the soluble protein. It is notable that glyceraldehyde 3-phosphate dehydrogenase (GAPDH), aconitase, voltage-dependent anion channels (VDACs), the ATP synthase alpha-chain, lactate dehydrogenase (LDH), beta-actin, and alpha-tubulin—which are either mitochondrial proteins or are known to interact with mitochondria—have been identified as oxidation targets in AD brains. This indicates that protein abundance may discriminate which protein targets are more susceptible to oxidative modifications. In other words, the decrease in the protein levels of several mitochondrial proteins in NDs may be due to their oxidation and precipitation in insoluble protein aggregates [85,86]. Again in the context of AD, the proteomics analysis of mitochondria isolated from peripheral blood lymphocytes has highlighted several proteins altered between controls and AD patients, grouped into four categories: (i) cellular energetics, including GADPH, LDH B-chain, and ATP synthase subunit beta; (ii) structural proteins, including annexin, beta-centractin, and myosin light polypeptide 6; (iii) cell signaling, including Rho GDP-dissociation inhibitor 2 (RhoGDI); and (iv) cellular defense, including thioredoxin-dependent peroxide reductase/peroxiredoxin III (PDXIII) [87]. In addition, reduced OXPHOS complexes I, III, and IV activity has been reported in platelets from AD patients and in postmortem brains [88].

### 4.3. Mitochondrial Proteomics in Parkinson’s Disease

The Italian proteomics community has also, in the context of PD, significantly contributed to the identification of mitochondrial pathways involved in the pathogenesis. The central role of mitochondrial dysfunction in PD pathobiology was first suggested by the discovery that mutation in genes encoding mitochondrial proteins is a cause of familial forms of PD [89,90]. Following this, a strong body of evidence was collected, supporting the role of mitochondrial dysfunction in the early pathogenesis of the sporadic forms of the disease also, which represent the majority of the cases. In addition to this, mitochondria-targeted toxins (e.g., MPTP and rotenone) were shown to cause Parkinsonian symptoms in humans. Based on these observations, PD animal models have been generated through either the specific knock-out or the pharmacological targeting of ETC complexes [91,92].

In a pioneering paper, Basso and co-workers compared protein extract from the substantia nigra of PD patients and controls, and identified forty-four proteins expressed in this mid-brain region by peptide mass fingerprinting, among which, nine showed changes in their abundance: L and M neurofilament chains were less abundant in PD, whereas peroxiredoxin II, mitochondrial complex III, the ATP synthase D-chain, complexin I, profilin, the L-type calcium channel delta-subunit, and the fatty-acid binding protein were more present in PD samples than controls [93]. These results were the first to suggest a possible potentiation mechanism of afferent signals to SN, upon degeneration of dopaminergic neurons.

De Iuliis and co-workers first highlighted common neurodegeneration mechanisms in PD and AD, related to mitochondrial function, by the analysis of SN from hemiparkinsonian rats, a PD animal model obtained through unilateral intranigral injection of 6-hydroxydopamine (6-OHDA) [94]. After protein separation and quantification by 2DE, they identified alpha-enolase and beta-actin as differentially expressed proteins in lesioned SN, compared to controls. These proteins were also found to be oxidized and modulated in AD.

Alberio and co-workers deeply investigated the alterations in the mitochondrial proteome due to dopamine exposure in the SH-SY5Y human neuroblastoma cell line, by two-dimensional electrophoresis and by shotgun proteomics [16]. The results highlighted the fragmentation of some mitochondrial proteins, suggesting a possible alteration of the activity of mitochondrial proteases, which are key enzymes involved in mitochondrial quality control. The possible aberrant activation of mitochondrial proteases in conditions of altered dopamine homeostasis was further investigated, using a degradomics approach, by Lualdi and co-workers, who first proposed neprilysin as a mitochondria-localized protease aberrantly activated by dopamine treatment in SH-SY5Y cells [95]. The recent development of terminomics approaches in proteomics, which allow the quantitative assessment of protease-generated fragments by their labeling, enrichment, and LC–MS/MS analysis, greatly improved our knowledge about mitochondrial proteases, their targets, and their role in NDs [96,97]. Indeed, mitochondrial proteases perform quality-control surveillance, by degrading misfolded and non-assembled polypeptides, and regulate the activity of specific substrates by mediating their processing steps. They are also directly involved in NDs (as shown for the m-AAA protease), and may regulate mitochondrial molecules, such as OPA1, which in turn are implicated in the pathogenesis of NDs [98].

## 5. Conclusions

The battle against neurodegenerative diseases is one of the biggest challenges that researchers worldwide must face. In the past, NDs have always been considered as uncurable disorders, for which treatments were aimed at ameliorating symptoms and patients’ quality of life. Nowadays, the efforts of the scientific community are mainly focused on the discovery of disease-modifying therapies, in the attempt to find a cure, or at least prevent the onset of the severest clinical manifestations. Targeted hypothesis-driven approaches are not fit for this purpose. Omics strategies, which consider the complexity of the disease system, represent the best weapon we have to defeat NDs.

The Italian proteomics community has contributed significantly to the knowledge of NDs, through the development and application of different proteomics strategies. The setting-up of protocols for the enrichment and quantitative analysis of PTMs (e.g., redox proteomics), the implementation of high-throughput platforms for biomarker discovery in biological fluids, the standardization of the procedures for the analysis of the mitochondrial proteome in health and disease states, and the development of bioinformatic tools for the network-based functional analysis of omics data, and their integration in a multi-omics approach, are some examples of the most recent advancements achieved in the study of NDs. These approaches represent powerful tools for the study of all complex diseases. Many pitfalls still exist in the proteomics field, especially in the context of biomarker research, where general rules for the standardization of the procedures (from sample collection to validation) are now being proposed and shared within the community. Additionally, technological improvements in the direction of high-throughput platforms, paralleled by cost-per-analysis reduction, are mandatory for the future employment of omics strategies in standard clinical procedures.

In this regard, multi-omics strategies are expected to return impactful results in NDs research: (i) the pathobiology of NDs will be investigated at the highest-possible level of complexity, unveiling molecular factors and pathways to be tested as candidate targets for therapy; (ii) novel, robust, and reliable panels of disease biomarkers will be identified, thus allowing patients to be diagnosed earlier, stratified based on their disease phenotype, and classified in groups based on their responsiveness to a specific treatment. The latter aspect is likely to be the most important one, since the integration of patient-specific proteomic and metabolomic profiles, for example, provides a comprehensive picture of how the patient is responding to a therapeutic intervention, in terms of both benefits and toxic effects. If such patient-specific omics strategies become routinely applied in clinics, we will eventually achieve the goal of personalized medicine.

## Figures and Tables

**Table 1:** Pathological mechanisms of MS, AD, and PD, recently unveiled by Italian proteomics studies.

ND	Pathological Feature	Reference
Multiple sclerosis	- Cortical damage severity, related to increased levels of proteins involved in complement/coagulation cascade, iron metabolism, and innate immune response. - High levels of cystatins in saliva, related to impairment of protein turnover and antioxidant mechanisms.	[10] [11]
Alzheimer’s disease	- Astrocytes alterations central in the early stages. Pathways related to astrocytes dysfunction: calcium signaling, RNA binding, and ribosomal dynamics. - Bioenergetics impairment (reduced glycolysis, increased ROS production, reduced oxygen consumption) in astrocytes. - Aberrant crosstalk among energy metabolism, mTOR signaling, and protein homeostasis in the brain.	[12] [13] [14] [15]
Parkinson’s disease	- Altered dopamine homeostasis, related to mitochondrial damage. - Impairment of vesicle trafficking, Rab GTPases activity, cell adhesion, cell migration, and cell signaling in primary skin fibroblasts.	[16] [17] [18]

**Table 2:** Outline of the most recent pathogenic PTMs discovered in NDs by Italian proteomics studies.

ND	PTM	Reference
Alzheimer’s disease	- Increased nitration (3-nitrotyrosine) of proteins involved in energy metabolism (PI3K, ATP synthase), cytoskeletal structure and signaling (ANXA2, DRP2), protein folding (HSC71), and antioxidant response (CAT). - Reduced O-GlcNAcylation of proteins involved in neuronal structure, protein degradation, and glucose metabolism (GAPDH, ENO1, MDH). - Increased poly-Ubiquitination of proteins involved in axonal growth, protein homeostasis, and transcriptional control under stress conditions in the brain (DRP2, HSP 90-β, eIF2α). - Increased PARylation of proteins involved in mitotic control (SMC3, ANAPC4), ubiquitination (NEDD4, USP10), and stress response (HSP70, DNAJC3, TAO3 kinase) in microglial cells. PARylation may be responsible for the transition of microglial cells to an inflammatory phenotype.	[36] [37] [38] [39]
Amyotrophic lateral sclerosis	- Decreased phosphorylation of proteins involved in muscular actin–myosin regulation downstream to overexpression of MyBP-H and decreased activity of ROCK and LIMK1 kinases.	[40]
Multiple sclerosis	- Increased sulfhydration of transthyretin, related to demyelination.	[41]
Parkinson’s disease	- Decreased phosphorylation of 14-3-3γ (direct interactor of LRRK2), related to neurite shortening.	[42]
